# Do Differing Levels of Boldness Influence the Success of Translocation? A Pilot Study on Red Squirrels (*Sciurus vulgaris*)

**DOI:** 10.3390/ani10101748

**Published:** 2020-09-26

**Authors:** Jack A. Bamber, Craig M. Shuttleworth, Matt W. Hayward

**Affiliations:** 1 School of Biological Sciences, Bangor University, Deiniol Road, Bangor LL572UW, Gwynedd, Wales, UK; Craig.Shuttleworth@rsst.org.uk (C.M.S.); matthew.hayward@newcastle.edu.au (M.W.H.); 2 School of Biological Sciences, University of Aberdeen, King’s College, Aberdeen AB24 3FX, UK; 3 School of Environment, Natural Resources and Geography, Bangor University, Deiniol Road, Bangor LL572UW, Gwynedd, Wales, UK; 4 Centre for African Conservation Ecology, Nelson Mandela University, Port Elizabeth 6013, South Africa; 5 School of Environmental and Life Sciences, University of Newcastle, University Drive, Callaghan, NSW 2308, Australia

**Keywords:** European red squirrel, *Sciurus vulgaris*, personality, boldness, translocation success, population restoration

## Abstract

**Simple Summary:**

As many species go extinct across the world, conservation initiatives seek to protect and boost wildlife populations, including through translocation programmes that involve the movement or release of captive or wild-caught individuals back into areas they have historically occupied. Captive-bred animals to be released into the wild can undergo behavioural testing to determine if the individuals have skills that would aid survival and increase the establishment of new populations. This research illustrates different levels of boldness in red squirrels and suggests selecting animals for release based on their boldness or timidity. Despite working with a low sample size in this pilot study, we observed trends suggesting that selecting individuals based upon a bold/timid scale of personality may improve future releases of red squirrels. By releasing timid animals first, the establishment of a population may be more successful, and this can be followed by releasing bold animals in later releases to enable faster distribution expansion of the population. This could result in improved success rates of restoration programmes, thus, lowering costs, improving the plight of biodiversity, and reducing early deaths of the released wildlife. Thus, we highlight a need for future research and collaboration within the translocation sector to assess personality.

**Abstract:**

Conservation translocations, including reintroductions, are practices that are vital to restoring biodiversity and ecosystem function within conservation schemes globally. Sadly, population translocations have had a poor success rate historically. At a time where biodiversity is constantly decreasing, improving translocation success is vital for future conservation schemes. Often, to improve success, the selection of individuals is based on genetic characteristics and behaviours linked directly to survival. Further development to improve selection is proposed within this paper using animal personality. The study took place opportunistically to test how personality, in particular an animal’s boldness/timidness, may influence a population restoration of red squirrels into the Ogwen Valley, North Wales. Despite frequent translocations, data on how boldness and timidness may affect the establishment of this species are low. Testing was performed on key survival behaviours and boldness/timidness pre-release. This was performed via video data collection and identification of key behaviours that could be used to identify boldness or behaviours that could be linked to reduced fitness once released. Encounters at different distance intervals were monitored post-release via camera trapping to identify if boldness/timidness may change the furthest encounter distance of focal animals away from their release site. Relationships between the period for an individual to reappear post-threat was significantly linked to boldness, with other behavioural results and the encounter distance also showing trends of a potential relationship. Our results indicate that bolder individuals have a higher chance of expressing behavioural traits that will increase exposure to risks and, therefore, reduce the likelihood of successfully establishing populations. However, the small sample size of this study means that further research is needed. We suggest that during early stages of conservation translocation programmes, personality testing for boldness should become common practice, and we recommend selecting timid individuals for an initial release to improve population establishment, with bolder individuals utilised later to expand population distribution.

## 1. Introduction

Reintroduction and reinforcement are forms of population restoration that aim to restore populations into areas where species or populations have become extinct, are reduced in number or fragmented [1,2,3,4,5,6]. All species bring specific roles to ecosystems, and the removal of any can result in severe negative effects on ecosystem function and balance [7]. Current changes in climate and human land-use are causing a loss of viable habitats for many species, rapidly increasing the occurrence of fragmented populations and extinctions [8]. Where in-situ conservation has failed to be successful or repopulation by remnant populations is not possible, the restoration of species through translocation is often the only way to rebuild native species populations, but it should not be used in the place of earlier conservation interventions [9]. This highlights the importance of reinforcement and reintroduction translocations to maintain and increase populations. Returning species and restoring their roles allow ecosystems to regain function and stability, which have been regularly observed when restoring keystone species [9,10].

Despite their conservation potential, translocations are often unsuccessful; some projects have mortality rates ranging from 85–100% of released individuals, resulting in complete failure or significantly lower founder populations and causing populations to fail to establish [11,12]. Key issues linked to early death in translocated animals are human–wildlife conflicts, faunal conflict/predation, stress, and an inability to avoid hostile environments [12]. With captive animals, many vital survival skills are not developed from birth, and, often, stereotypic behaviours are developed instead, with negative effects on survival instincts that allow the factors above to have a heavy influence [13]. In great ape translocations, extensive behavioural testing is recommended to select animals with an increased chance of survival by selecting animals that exhibit natural behaviours and avoiding those who express behaviours that have no benefit once released, such as any stereotypic behaviours [14]. Stereotypic behaviours are developed through a lack of stimulus in captivity and are problematic as the animals who are released back into the wild waste time and resources without gain, resulting in reduced fitness and/or death if they are unable to adapt their behaviour to suit new environmental needs [15,16]. Similar practices should, therefore, be implemented for all conservation translocations. Utilising the assessment of animal personality is a potential way of selecting individuals that are capable of avoiding hostile situations and more able to develop relevant survival skills, making it a multiapplicable approach to selection. This, in turn, would increase the likelihood of survival and population establishment in translocations. 

The key personality trait that is likely relevant to population restoration is the scale of boldness [17,18]. When quantifying if an animal is bold or timid, an assessment of behaviours and responses to situations is performed to determine at what rate an animal responds in what is identified as a bold or timid way [19]. The benefits of assessing animal personality have been seen in assessing both the welfare and fitness of fauna [20,21], highlighting the potential to utilise personality to improve population restoration. Bolder individuals have larger home ranges, which may increase the chance of encountering risky situations, and this is combined with bolder individuals being less likely to avoid risk, thereby decreasing their survival chances [21]. Conversely, more timid individuals will directly avoid potentially risky situations and, thus, avoid predation, injury and conflict [22]. This difference in an individual’s ability to assess and avoid risk could be used to improve translocation success rates. 

Boldness may have ramifications when captive-bred animals are used in translocations. There is potential for bolder individuals to be more comfortable on show in captivity without negative stress-related consequences, while timid individuals are potentially more suited for the stresses of release, allowing both sides of the bold/timid scale to be of benefit for conservation and welfare [11,23]. With an individual’s ability to avoid risk heavily linked to survival, the ability to select individuals that would avoid risk would be of high value for translocation. 

**Figure 1:**
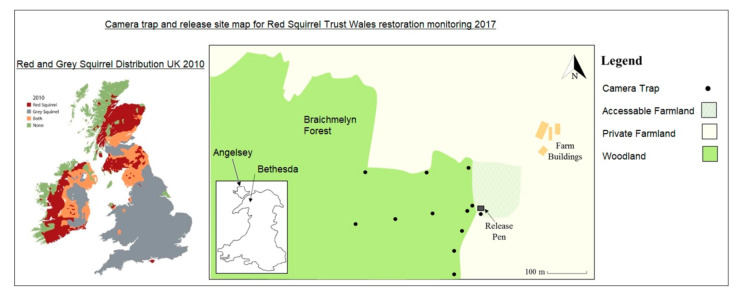
Camera-trap locations within the forest area of the Ogwen valley alongside current red squirrel distribution across the UK. Traps are marked with ●, and the original release pen is marked with a square; traps were set 100 m apart from each other, away from the release pen. British red squirrel distribution shown in red, grey squirrel distribution in grey, shared zones in orange and green showing no population of either species [31].

Red squirrels, *Sciurus vulgaris,* are currently abundant across most of their historic range throughout mainland Eurasia and are listed on the IUCN Red List as Least Concern [24]. However, across Britain, their numbers are dramatically reduced as populations are fragmented compared to their historical distribution (Figure 1) [25]. Regional extinctions and frequent reduction in red squirrel population numbers elsewhere in Britain are due mainly to the range expansion of invasive eastern grey squirrels, *Sciurus carolinensis*, introduced in the late 1800s [26,27]. Ecologically, red squirrel*s* are important for forest tree regeneration due to seed caching and are less impactful to forestry and tree growth due to reduced stripping of native tree bark compared to grey squirrels [28,29]. Economically, red squirrels are the key flagship species that is able to promote reintroductions and conservation schemes and generate ecotourism due to the positive social impact red squirrels provide as a species that is well-loved by the public [30]. Due to these factors, there are numerous ongoing reintroductions of red squirrels, highlighting the need for improved understanding in ways to improve reintroduction success. A previous study based on American red squirrels, *Tamiasciurus hudsonicus*, found that personality type had significant effects on the ability of wild red squirrels to assess risk and successfully utilise their habitat [22]. This suggests that differing degrees of boldness/timidness may also have an impact on the survival of Eurasian captive red squirrels that are utilised in reintroduction and translocation programmes and may also influence their ability to assess risks.

This pilot study was performed opportunistically to evaluate the potential use of boldness within translocation during a reinforcement translocation of red squirrels into the Braichmelyn Forest, Ogwen Valley, North Wales, UK, performed by the Red Squirrel Trust, Wales (Figure 1). A reinforcement translocation moves animals from one location or captivity to a new location with the aim of increasing genetic variability and distribution within reduced or fragmented existing populations [6]. This conservation project aimed to expand the mainland range of red squirrels in Gwynedd county after natural dispersal occurred from the island of Anglesey near the Bangor area via assumed corridors created by low tides and transport bridges. At the time of this release programme in 2016, there were no known red squirrels in the release area and the local large pine plantation was deemed a suitable ecosystem to maintain a red squirrel population. Working with a small sample size, this pilot study aimed to assess links between boldness and factors that may influence survival post-release to improve the selection of individuals for future red squirrel translocations. 

This study aimed to analyse the behaviours of individuals prior to their release and relate this to their post-release movements to aid in the selection of individuals for translocation. We predicted that (1) individuals with a bolder personality type would travel further from the release site, placing them at greater risk of encountering threats and dying, (2) individuals with a bolder personality type would have longer times of latency before fleeing and shorter reappearance times after fleeing when presented with a risk stimulus, and (3) individuals with bolder personality types will perform larger amounts of stereotypy, linked with decreased success within the translocation.

## 2. Materials and Methods

Prior to release, squirrels were housed in a pre-release pen (dimensions 4 × 4 m^3^), housing up to four individuals at a time. Internally, the pen replicated the environmental conditions of the release site, utilising mosses, branches and shrubs collected from the adjacent woodland, with the addition of nest boxes and feeding platforms. The release took place on private wooded farmland in Bethesda, North Wales, alongside a larger commercial conifer plantation that is suitable for sustaining a red squirrel population, Braichmelyn Forest. Local predators that may pose a risk to red squirrels ranged from the common buzzard, *Buteo buteo,* to the red fox, *Vulpes vulpes*, which were identified through camera trapping programmes in nearby woodlands in Gwynedd county. Despite their captive-bred history, the study animals provided by Pensthorpe Conservation Trust had not been habituated to human presence as they were juvenile animals that had been kept off-show. Subjects were held in pre-release pens for three weeks and fed via scatter feeding, without prolonged human presence, to encourage natural behaviour, then released. The provided diet was a mixture of nuts and seeds as well as fruits and berries. Release groups were kept together in trios and were allowed an adjustment period of one week within the release pen prior to behavioural data collection. Post-release, individuals had access to the pen for up to three weeks to allow adjustment, then the pen was closed and utilised for the next release group of three. During the research period of this pilot study, two release groups of three individuals were released and studied (Table 1).

Camera trap and feeder networks were erected within the forest area to monitor the movement and status of individuals. A total of 12 cameras (Bushnell Natureview, Bushnell, Wales, UK) were set from the release site at 100 m intervals into the forest area (Figure 1). Feeders were provided for the released individuals to assist their survival, meet legislative requirements and to encourage squirrel detection at camera trap sites for monitoring. Each of the 12 cameras had a feeder within view, feeders and cameras were checked and replenished with sunflower seed weekly over three months (December 2016–March 2017).

Behavioural observations were collated via two cameras (GoPro Hero3+, GoPro, Cheshire, UK) within the release enclosure. Cameras were utilised to remove the behavioural effects that observers can have upon individuals when present, allowing normal behaviour to be observed [32]. Cameras recorded at sunrise for three hours to encompass a peak activity period due to red squirrels being crepuscular. A total of 80 hours of video footage was collected for both release groups over a two-week period. This video footage was utilised to analyse both boldness/timidness and behaviours negatively associated with survival, such as stereotypy. Individual animals were identified by pelage markings during behavioural testing (Table 1). Flight time was defined as the amount of time the squirrel remained present upon the arrival of an observer (50 m away) and this arrival was utilised as a novel stimulus. Reappearance time was recorded from video footage once the researcher had left the site. Flight and reappearance were defined by the amount of time for a focal animal to hide or emerge from view (likely into a nest box), recorded in seconds. 

Video footage also assessed pre-release personality on the bold/timid scale by identifying the frequency of bold and timid behaviours observed. Behaviours were identified using a predefined ethogram (Supplementary Materials Table S1), with bold and timid behaviours identified and developed similarly to Bremner-Harrison et al. (2004) [21], who defined bold and timid behaviours in a translocation of swift foxes *Vulpes velox*. This was applied alongside personal observations of the sample animals and previously produced squirrel behaviour ethograms to create the ethogram, focused on red squirrels, for use within this study. Behaviour scores ranged from +1 for boldness to −1 for timidness. Boldness was created by the total sum of timid or bold behaviours observed in each individual. The activity was filmed over a fourteen-day period; the minimum period that a squirrel was detected and able to be studied was eight days out of fourteen. To avoid bias, each animal had eight days of observations used for score creation; these were selected at random from available recordings for all five study animals. Then, the behaviours observed, defined as bold or timid (see ethogram; Supplementary Materials Table S1), were utilised to score that animal on that day. The boldness scores created ranged from −10 (timid) to +62 (bold; Table 1). Four behaviours, found in the literature to reduce survival chance, were then compared to bold/timid scores: fleeing latency, reappearance post-threat, stereotypic behaviour, and total activity. Each of the four behaviours was found to be normally distributed.

The study monitored the last known fates of individuals from the camera trap network, with recordings of date, time and location of 13,694 pictures over the 12-week sampling period. This allowed for the estimation of survival. Radio telemetry tracking was not possible due to animals being undersized; welfare collars had to weigh <5% of each squirrel’s mass, and, therefore, confirmed deaths were not possible to record. The total research period from the arrival of focal animals at release pens to the end of field sampling was approximately 20 weeks. Survivorship was analysed via Kaplan–Meier analysis with staggered entry, utilising the last known sighting of each squirrel [33]. The maximum distance that camera traps identified individual release animals was also collected through the camera trap network. This was analysed alongside personality scores in order to assess any relationship between boldness and dispersal. 

Statistical analyses were carried out using R Studio (R 3.3.3, Rstudio, Boston, MA, USA). Data conformed to normality assumptions of parametric statistics. To assess how boldness may relate to the expression of behaviours linked to survival and risk-taking, linear regressions were performed. The regressions analysed the relationship of the boldness/timidness of each animal, and the mean time periods these behaviours were expressed. The maximum distance that each individual moved away from the release site was related to bold/timid scores to identify any relationship between the two.

## 3. Results

### Key Results

Two individuals died during the research period (December 2016–March 2017), one prior to release and one post-release. The pre-release death occurred early after arrival at the release site; the individual was found in a nest box with a head injury. Postmortem testing revealed traces of listeria and *Escherichia coli*. The second individual died post-release and was found in a nest box with dark faecal staining of the perineum. A postmortem identified this to be linked to enteritis as a possible cause of death. All other individuals were still alive at the end of sampling. The individual that died within the release pen, prior to release, was excluded from statistical analysis. 

There was no relationship found between the mean time before fleeing and personality (R^2^ = 0.2929, n/df = 5, *p* = 0.2016,). There was a significant relationship between reappearance time and personality score (R^2^ = 0.826, n/df = 5, *p* = 0.021). Activity rates of individuals were tested for relationship with boldness scores. No significant relationship occurred between personality and periods of activity (R^2^ = −0.325, n = 5, *p* = 0.897).

**Figure 2:**
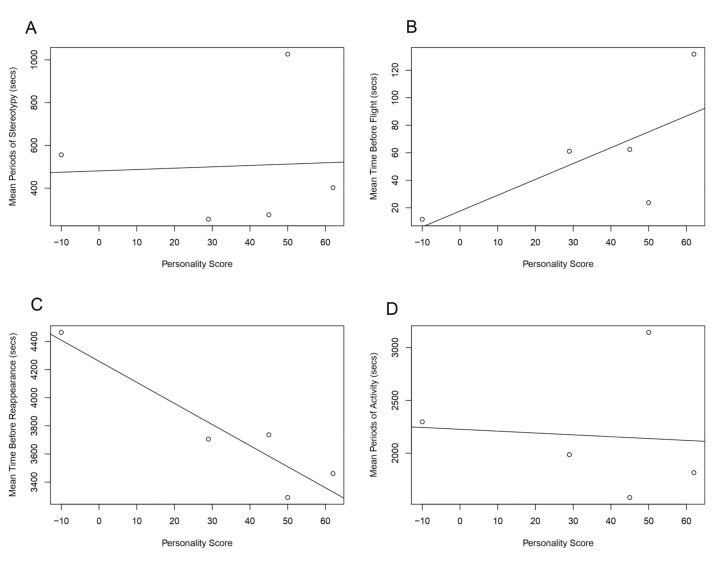
Linear regressions representing relationships between the mean expressions of key behaviours and personality scores, performed on a translocated group of red squirrels into the Braichmelyn forest, 2017: (**A**) Stereotypy(R^2^ = −0.3292, n/df = 5 , *p* = 0.9292), (**B**) flight (R^2^ = 0.2929, n/df = 5, *p* = 0.2016), (**C**) reappearance (R_2_ = 0.8258, n/df = 5, *p* = 0.0209), and (**D**) activity (R^2^ = -0.3246, n/df = 5, *p* = 0.8973).

Two forms of stereotypy were identified in individuals at differing rates; this was seen in all sampled animals at varying scales. This was tested for a relationship with boldness. There was no significant relationship between mean periods of stereotypy and personality (R^2^ = −0.3292, n/df = 5, *p* = 0.9292; Figure 2). 

**Figure 3:**
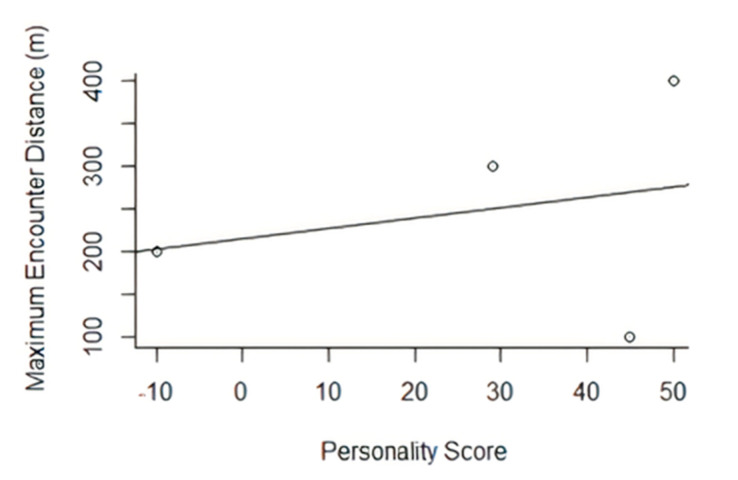
A linear regression showing the potential relationship between personality score and maximum encounter distance performed on a translocated group of red squirrels into the Braichmelyn forest in 2017 (R^2^ = −0.4014, n/sf = 4, *p* = 0.744).

The maximum distance an individual was sighted from the release site was not related to personality score (R^2^ = −0.4014, n/df = 4, *p* = 0.744; Figure 3).

## 4. Discussion

**Figure 4:**
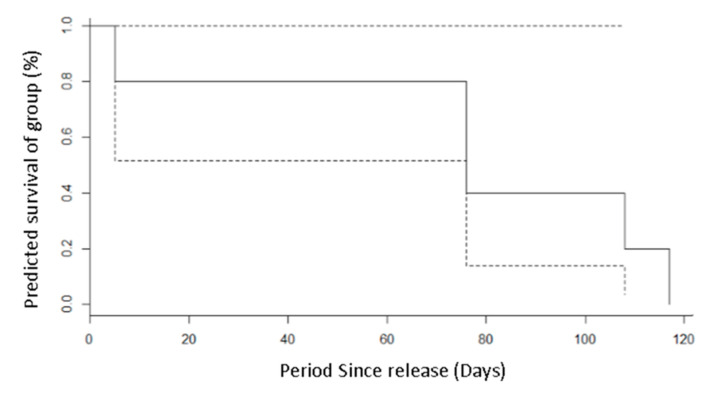
Kaplan–Meier survivorship curve showing the survival and last known points of capture Figure 2017. 95% confidence represented by the dashed line, with survival represented by the solid line.

Despite the low sample size preventing a direct assessment of boldness and timidness with survival, the individual sighted the latest, nearly 120 days post-release, was the most timid, as seen in (Figure 4). The squirrels released during this conservation programme have bred successfully since, indicating the establishment of a Braichmelyn Forest red squirrel population (CS *pers. obs.* latest sighting 1 September 2020). Our work shows that captive-bred Eurasian red squirrels appear to have different levels of bold/timid personality and that high levels of boldness significantly affects their reappearance rate when disturbed, potentially influencing their ability to avoid predation and establish populations post-release. Although the sample size was too small to detect differences in survivorship, lowered survival is likely to occur given the increased risks that other bolder individuals face during translocations and their decreased ability to avoid them effectively. It should be highlighted that due to the small sample caused by the opportunistic nature of the study, only one animal was identified as fully timid. This presents a potential bias towards bold animals; however, if boldness is viewed as a scale, individual differences could still be seen, with some bold animals being bolder than others. With a larger sample size of more personalities, a longer sampling period and more confirmations of fate, the link between personality and survival of released fauna may be demonstrated in a more definitive way or may adversely disprove this theory. Alternatively, further analysis with more timid individuals may reveal positive benefits of higher boldness in certain release programmes. With release programmes of red squirrels often being performed in low numbers and funding often limited, a collaboration between multiple small release programmes such as this may be the key to finding significant links. 

An individual’s choice of when to flee and re-emerge is based on its assessment of risk, and both are heavily linked when avoiding predation [34]. This is due to individuals who delay in fleeing having an increased chance of being predated. Equally, those who are early to reappear from safety may be faced with the same predator they had initially escaped [34,35]. The ability to successfully adapt to being released and the new threat of predators is a key factor in how efficient individuals are at surviving when no longer within captivity [36]. This is potentially linked to boldness, with trends pointing towards bolder individuals having an increased chance of being predated due to flight latency and reappearance times lowering their potential to survive and establish post-release. However, due to the low sample size, causation cannot be concluded. This adds a dynamic to the selection of individuals for translocation based on boldness, with an increased chance of predation on bolder individuals. Within the current literature, the need to look at an animal’s ability to avoid predation through antipredator training prior to translocations has been highlighted as a key step in improving release and may be linked to individual personality [37]. This shows a relationship with the results and baseline theory discussed in this project.

During behavioural testing, two stereotypical behaviours were identified (i.e. those behaviours with no benefit other than a lack of stimulus and boredom) [38]. The two stereotypical behaviours seen were pacing side to side in the corner of the cage and repetitive banging of paws up and down onto the wire mesh of the cage. The amounts of stereotypy performed by individuals varied. This was compared with bold/timid personality types in Figure 2 to assess any possible link. There was a positive trend between stereotypy and boldness but not significantly. Stereotypy has been linked heavily with a decrease in fitness of translocated captive fauna [15,39]. This factor is, therefore, worth considering when selecting individuals, and the potential link between boldness and stereotypy should be looked at further in a larger study.

There was a positive trend between boldness and the furthest-encounter distance of each individual. This was not significant within the study but, at an individual scale, the boldest released individual was found at the furthest point from the original release site. Movements of individuals are vital for population restoration and may vary between specific release programmes. In many cases within a translocation, movement patterns of the released individuals are key for primary establishment, then dispersal and merging with other localised populations of the species, improving the genetic variation and distribution of the focal species by rejoining fragmented populations [4,5,6]. Therefore, in this case, it would be considered of benefit to choose bolder individuals to achieve this movement and interaction with fragmented groups. 

In complete reintroduction translocations, many species would benefit by remaining closer to their release site to avoid conflict and increase the chance of survival and establishment [40,41]. This would be particularly relevant for releases of other native species that are vulnerable to human–wildlife conflict through negative association and persecution, such as the pine marten *Martes martes* [40,41,42]. By choosing more timid individuals, they may stay closer to the release site due to decreased bold explorative behaviour, increasing the ability of wildlife managers to track and control where individuals may go. By choosing release sites far enough away from human populations, whilst selecting more timid animals, conservation practitioners may remove potential human conflict. Some animals are solitary or territorial and may force competitors out of new territory once released, impacting home ranges and dispersal through intraspecific competition for resources [43]. Selecting timid individuals first may also prevent conflict in the early stages of release due to less movement, allowing easier establishment, but may lead to increased dispersal over time if bolder individuals expand their range and force a more timid individual to move away or if multiple animals are released in close proximity. Having lower ranges of movement by selecting timid individuals may also help individuals to avoid transport networks, with many fatalities after release being caused by collisions with vehicles [44]. By choosing individuals based on their boldness, selection could easily be made to suit the location and premise of any population restoration. Further assessment of movement is recommended with radio tracking of individuals to collect more exact data on home range and movement patterns to find significant relationships with personality traits. This would further assess how boldness is a predictor of movement and how applicable it could be to the selection of individuals for conservation translocation. Collaboration between multiple release programmes, collating and assessing bold/timid personality in individual species, would also allow further assessment of how this may influence survival within translocations.

Even with the low sample size within this study, the potential for a link between boldness and a decreased survival chance is viable. With a significant relationship occurring in reappearance time, a factor linked to the survival of prey species, and trends being seen in the direction of potentially negative effects for survival behaviour, the chances of released-individual personality affecting translocation success are present. The use of personality assessment when selecting individuals for population restoration would be of benefit to fit the needs and restraints of different release programmes to ensure the individuals are established in areas that are suitable to specific reintroduction plans. The use of personality could essentially help to prevent negative interactions with man-made and natural threats by providing an automatic advantage to some individuals without conditioning, thereby increasing survival chance. With deaths within population restorations being high, as stated throughout, any possible way of decreasing mortality and aiding the selection of fauna to increase the establishment of populations should be considered and researched further.

## 5. Conclusions

It is clear from the literature that an individual’s ability to assess and avoid risk is key to their ability to establish and survive. The results collated from this work show bolder individuals seem to have a higher chance of expressing behavioural traits that will decrease their ability to avoid risk and, therefore, may prevent the establishment of new populations. It is therefore suggested from this work that wherever possible during early stages of conservation translocation, personality assessments should be performed to calculate individual boldness. In future studies, a collaboration for more timid individuals to be selected for early-stage release will potentially improve initial success, with bolder individuals utilised later to expand population distribution. 

## Figures and Tables

**Table 1:** Profiles of red squirrels studied during a translocation release in Braichmelyn Forest in 2017 showing individual boldness scores. Higher positive scores and lower negative scores are aligned with bolder or more timid individuals, respectively. The larger the -/+ score indicates how bold or timid an individual is.

Individual	Release Group	Sex	Bold/Timid Score	Pelage Markings
1.1	1	F	50	Small, grey/brown patch on left flank, remaining fur is light orange. White snout grey/orange face.
1.2	1	M	−10	Deep orange colouration, sleek flat tail hair, grey colouration on flanks, across back and rear. Entire back is almost grey.
2.1	2	F	62	Dark orange with grey markings down flanks, white spot visible on the back of head. White nose, dark orange face.
2.2	2	M	29	Dark orange with darker markings on flanks, dark grey face, grey streak down tail. Large white spot on right-hand side of its back.
2.3	2	F	45	Light orange fur, almost fully white face, Body almost completely orange, slight grey markings on flanks.

## Supplementary Materials

The following are available online at https://www.mdpi.com/2076-2615/10/10/1748/s1, Table S1: A copy of the ethogram utilised in behavioural assessment.

## References

[1] Kleiman D.G. (1989). Reintroduction of Captive Mammals for Conservation. Bioscience.

[2] Seddon P.J., Armstrong D.P., Maloney R.F. (2007). Developing the Science of Reintroduction Biology. Conserv. Biol..

[3] Armstrong D.P., Seddon P.J. (2008). Directions in reintroduction biology. Trends Ecol. Evol..

[4] Griffith B., Scott J.M., Carpenter J.W., Reed C., Gewirtz A., Calabretta B. (1989). Translocation as a species conservation tool: Status and strategy. Science.

[5] Emslie R., Amin R., Kock R. (2009). Guidelines for the In-Situ Re-Introduction and Translocation of African and Asian Rhinoceros.

[6] IUCN, SSC (2013). Guidelines for Reintroductions and Other Conservation Translocations.

[7] Jones C.G., Lawton J.H., Shachak M. (1994). Organisms as Ecosystem Engineers. Oikos.

[8] Hayward M.W. (2011). Using the IUCN Red List to determine effective conservation strategies. Biodivers. Conserv..

[9] Knapp A.K., Blair J.M., Briggs J.M., Collins S.L., Hartnett D.C., Johnson L.C., Towne E.G. (1999). The Keystone Role of Bison in North American Tallgrass PrairieBison increase habitat heterogeneity and alter a broad array of plant, community, and ecosystem processes. Bioscience.

[10] Hayward M.W., Somers M. (2009). Reintroduction of Top-Order Predators: Using Science to Restore One of the Drivers of Biodiversity.

[11] Teixeira C.P., De Azevedo C.S., Mendl M., Cipreste C.F., Young R.J., Mendl M. (2007). Revisiting translocation and reintroduction programmes: The importance of considering stress. Anim. Behav..

[12] Fischer J., Lindenmayer D. (2000). An assessment of the published results of animal relocations. Biol. Conserv..

[13] Mason G. (2006). Stereotypic Behaviour in Captive Animals: Fundamentals and Implications for Welfare and Beyond. Stereotypic Animal Behaviour: Fundamentals and Applications to Welfare.

[14] Walkup C., Rodrigues M., Unwin S., Travis D., Stoinski T. (2007). Best Practice Guidelines for the Re-Introduction of Great Apes.

[15] Vickery S.S., Mason G.J. (2003). Behavioral persistence in captive bears: Implications for reintroduction. Ursus.

[16] Shepherdson D. (1994). The role of environmental enrichment in the captive breeding and reintroduction of endangered species. Creative Conservation.

[17] Sneddon L.U. (2003). The bold and the shy: Individual differences in rainbow trout. J. Fish Biol..

[18] Bremner-Harrison S., Prodöhl P., Elwood R.W. (2004). Behavioural trait assessment as a release criterion: Boldness predicts early death in a reintroduction programme of captive-bred swift fox (*Vulpes velox*). Anim. Conserv..

[19] Dingemanse N.J., Kazem A.J., Réale D., Wright J. (2010). Behavioural reaction norms: Animal personality meets individual plasticity. Trends Ecol. Evol..

[20] Creighton E., Pankhurst S. Animal personality and animal welfare. Proceedings of the 8th Annual Symposium on Zoo Research 2007.

[21] Smith B.R., Blumstein D.T. (2008). Fitness consequences of personality: A meta-analysis. Behav. Ecol..

[22] Boon A.K., Reale D., Boutin S. (2007). The interaction between personality, offspring fitness and food abundance in North American red squirrels. Ecol. Lett..

[23] Moberg G.P., Mench J.A. (2000). The Biology of Animal Stress: Basic Principles and Implications for Animal Welfare.

[24] Shar S., Lkhagvasuren D., Bertolino S., Henttonen H., Kryštufek B., Meinig H., IUCN (2016). *Sciurus vulgaris*.. IUCN Red List of Threatened Species.

[25] Barratt E.M., Gurnell J., Malarky G., Deaville R., Bruford M.W. (1999). Genetic structure of fragmented populations of red squirrel (*Sciurus vulgaris*) in the UK. Mol. Ecol..

[26] Reynolds J.C. (1985). Details of the Geographic Replacement of the Red Squirrel (*Sciurus vulgaris*) by the Grey Squirrel (*Sciurus carolinensis*) in Eastern England. J. Anim. Ecol..

[27] Okubo A., Maini P.K., Williamson M.H., Murray J.D. (1989). On the spatial spread of the grey squirrel in Britain. Proc. R. Soc. London. Ser. B Biol. Sci..

[28] Kenward R.E., Parish T. (2009). Bark-stripping by Grey squirrels (*Sciurus carolinensis*). J. Zool..

[29] Hurly T.A., Robertson R.J. (1987). Scatterhoarding by territorial red squirrels: A test of the optimal density model. Can. J. Zool..

[30] Bowen-Jones E., Entwistle A. (2002). Identifying appropriate flagship species: The importance of culture and local contexts. Oryx.

[31] Shuttleworth C. (2010). Red Squirrel Distribution in the British Isles 1946–2010.

[32] Hosey G.R. (2000). Zoo animals and their human audiences: What is the visitor effect?. Anim. Welf..

[33] Pollock K.H., Winterstein S.R., Bunck C.M., Curtis P.D. (1989). Survival Analysis in Telemetry Studies: The Staggered Entry Design. J. Wildl. Manag..

[34] Cooper W.E., López P., Martín J., Pérez-Mellado V. (2012). Latency to flee from an immobile predator: Effects of predation risk and cost of immobility for the prey. Behav. Ecol..

[35] Jennions M.D., Backwell P.R., Murai M., Christy J.H. (2003). Hiding behaviour in fiddler crabs: How long should prey hide in response to a potential predator?. Anim. Behav..

[36] Griffin A.S., Blumstein D.T., Evans C.S. (2000). Training Captive-Bred or Translocated Animals to Avoid Predators. Conserv. Biol..

[37] Greggor A.L., Price C.J., Shier D.M. (2019). Examining the efficacy of anti-predator training for increasing survival in conservation translocations: A systematic review protocol. Environ. Évid..

[38] Mason G., Rushen J. (2008). Stereotypic Animal Behaviour: Fundamentals and Applications to Welfare.

[39] Jule K.R., Leaver L., Lea S.E.G. (2008). The effects of captive experience on reintroduction survival in carnivores: A review and analysis. Biol. Conserv..

[40] Villafuerte R., Viñuela J., Blanco J.C. (1998). Extensive predator persecution caused by population crash in a game species: The case of red kites and rabbits in Spain. Biol. Conserv..

[41] Treves A., Wallace R.B., Naughton-Treves L., Morales A. (2006). Co-Managing Human–Wildlife Conflicts: A Review. Hum. Dimens. Wildl..

[42] Bamber J.A., Shuttleworth C.M., Hayward M.W., Everest D.J. (2020). Reinstating trophic cascades as an applied conservation tool to protect forest ecosystems from invasive grey squirrels (*Sciurus carolinensis*). Food Webs.

[43] Schradin C. (2004). Territorial defense in a group-living solitary forager: Who, where, against whom?. Behav. Ecol. Sociobiol..

[44] Calenge C., Maillard D., Invernia N., Gaudin J. (2005). Reintroduction of roe deer Capreolus capreolus into a Mediterranean habitat: Female mortality and dispersion. Wildl. Biol..

