# Supplementary material for: Do Differing Levels of Boldness Influence the Success of Translocation? A Pilot Study on Red Squirrels (Sciurus vulgaris)
Source: Animals (Basel). 2020 Sep 26;10(10):1748. doi: 10.3390/ani10101748 (PMC7599904; doi:10.3390/ani10101748)
Supplement: Supplementary file 1 [file animals-10-01748-s001.pdf]

animals

Supplementary Materials: Do Differing Levels of Boldness Influence the Success of Translocation? A Pilot Study on Red Squirrels (*Sciurus vulgaris*)

Jack A. Bamber [1,2,*], Craig M. Shuttleworth [1] and Matt W. Hayward [1,3,4,5]

Table S1. A copy of the ethogram utilised in behavioural assessment.

Behaviour:	Description:
Sub class (Bold)	
1. Resting relaxed	Lay or sat possibly with eyes closed in a more relaxed stance. No scanning of area.
2. Eating relaxed	Sat eating without any pauses to scan area.
3. Exploring/Moving around pen relaxed	Move around pen sniffing ground concentrating on examining area.
4. Chasing internal conspecific	Engage in sudden running after another individual within the pen around the enclosure.
5. Chasing external conspecific	Engage in sudden running and interaction with an already released squirrel outside of enclosure.
6. Chasing internal heterospecific	Engage in a chase/charge towards one of the small birds which have access into enclosure.
7. Chasing external heterospecific	Engage in chase behaviour to a non-squirrel external of cage, often performed on crows.
8. Bold approach conspecific	Quickly and rapidly approach another squirrel within the enclosure.
9. Relaxed reception of bold approach	When approached, squirrel will respond calmly with no sudden jumps or flight.
10.Agressive behaviour towards Conspecific (Fight)	Engage in fight behaviour, the individual who engages this behaviour.
11. Jumping and leaping. High risk	Jump from the top of cage or from high areas towards the central bush or floor.
12. Lack of flight when provoked	Receive an alarm call, approach by large heterospecific or hear a predatory bird call, but remain unfazed.
13.Dominance/Threat display	Jump up and down, alarm calling often upon arrival of observer, performed often instead/before flight
Sub class (Timid)	
1.Prolonged scouting from nest box	Peaks head out of nest box to scan area. Record whether animal chooses to leave or to stay.
2.Sudden stop and fearful scan of area	Individual will stop whatever general behaviour it is doing suddenly, raise head or sit upright, and perform scan.
3.Unprovoked flight	Suddenly run from what it is doing towards a nest box, no visible cause or sound.
4.Provoked flight	Flee often towards a nest box, visible sign/sound such as a predatory bird call.
5. Running away from a conspecific external	Run from/be chased by another individual already released on the outside of enclosure.
5. Running away from a conspecific internal	Run from/be chased by another individual internal of enclosure.
6. Fearful response to conspecific approach	Will respond to an approach by another individual with a sudden jump or flight response
7. Cautious alert Resting	Will be sat resting but upright alert and scanning area regularly.
8. Cautious alert Eating	Eating but will often stop to scan the area.
9. Cautious alert Exploration	Exploring pen but will often stop to look and scan.
10. Cautious watching conspecific	Watching another individual in a tense position, twitching tail etc.
11. Cautious watching external of cage	Sat watching outside of cage still and twitchy, often followed by sudden flight.
Sub class (Stereotypic)	
Stereotypic banging on side of pen/Mesh	Individual sits at edge of pen moving up and down from waist banging on cage.
Stereotypic running at edge of pen (Pacing).	Individual runs back and two alongside of pen for long periods of time, most common in corner.
Sub class (Undefined)	
1. Out of sight.	Individual not visible.
2. Chewing or scratching objects.	Perform biting and scratching on boxes, cage, floor and branches.
3. Hanging from cage	Squirrel climbs cage and hangs from roof or side of mesh often scanning the area.
4.Flipping off the side of cage	Squirrel will run at cage and flip itself off the side of cage (Stereotypic)
5.Stretch	Animal stops and extends extremities out.
6. Locates food	Walking around enclosure stops over food source and picks it up
7. Eats food	Sit's and eats food, may takes food into nest box to consume.
8.Burys food	Takes food found and buries it in the ground.
9. Mating	Attempted or successful mounting of another squirrel, note if fighting occurs and who initiates, mating and fighting.
10. Scanning	Looks up on all fours and twitches tail and looks around area.
11. Excretion	Release faeces.
12. Urination	Release urine.
13. Running	Perform locomotion quickly from one area to another.
14. Walking	Perform locomotion slowly to one area to another.
15. Jumping/Leaping	Jump from one spot to another.
16. Climbing	Scale an area of the pen/branches.
17. Sniffing	Move around face on floor seeking scents.
18. Groom	Perform rubbing of fur on its body, cleaning dirt and debris.
19. Rear	Rise up from a walking position.
